# The Role of IKKβ in Venezuelan Equine Encephalitis Virus Infection

**DOI:** 10.1371/journal.pone.0086745

**Published:** 2014-02-19

**Authors:** Moushimi Amaya, Kelsey Voss, Gavin Sampey, Svetlana Senina, Cynthia de la Fuente, Claudius Mueller, Valerie Calvert, Kylene Kehn-Hall, Calvin Carpenter, Fatah Kashanchi, Charles Bailey, Soren Mogelsvang, Emanuel Petricoin, Aarthi Narayanan

**Affiliations:** 1 National Center for Biodefense and Infectious Diseases, George Mason University, Manassas, Virginia, United States of America; 2 Center for Applied Proteomics and Personalized Medicine, George Mason University, Manassas, Virginia, United States of America; 3 Serpin Pharma LLC, Manassas, Virginia, United States of America; CEA, France

## Abstract

Venezuelan equine encephalitis virus (VEEV) belongs to the genus *Alphavirus*, family *Togaviridae*. VEEV infection is characterized by extensive inflammation and studies from other laboratories implicated an involvement of the NF-κB cascade in the *in vivo* pathology. Initial studies indicated that at early time points of VEEV infection, the NF-κB complex was activated in cells infected with the TC-83 strain of VEEV. One upstream kinase that contributes to the phosphorylation of p65 is the IKKβ component of the IKK complex. Our previous studies with Rift valley fever virus, which exhibited early activation of the NF-κB cascade in infected cells, had indicated that the IKKβ component underwent macromolecular reorganization to form a novel low molecular weight form unique to infected cells. This prompted us to investigate if the IKK complex undergoes a comparable macromolecular reorganization in VEEV infection. Size-fractionated VEEV infected cell extracts indicated a macromolecular reorganization of IKKβ in VEEV infected cells that resulted in formation of lower molecular weight complexes. Well-documented inhibitors of IKKβ function, BAY-11-7082, BAY-11-7085 and IKK2 compound IV, were employed to determine whether IKKβ function was required for the production of infectious progeny virus. A decrease in infectious viral particles and viral RNA copies was observed with inhibitor treatment in the attenuated and virulent strains of VEEV infection. In order to further validate the requirement of IKKβ for VEEV replication, we over-expressed IKKβ in cells and observed an increase in viral titers. In contrast, studies carried out using IKKβ^−/−^ cells demonstrated a decrease in VEEV replication. *In vivo* studies demonstrated that inhibitor treatment of TC-83 infected mice increased their survival. Finally, proteomics studies have revealed that IKKβ may interact with the viral protein nsP3. In conclusion, our studies have revealed that the host IKKβ protein may be critically involved in VEEV replication.

## Introduction

The New World alphavirus VEEV belongs to the family *Togaviridae* [1]–[5]. VEEV is endemic to South America and has extended to the southern regions of the United States [3], [5]. The re-emergence of VEEV in Venezuela and Colombia in 1995 resulted in 75,000–100,000 human cases [5], [6]. VEEV, a zoonotic pathogen, is a mosquito-borne virus first isolated and characterized serologically in 1938 [1], [4], [6], [7]. Apart from mosquito bites, humans can also be infected via the aerosol route as determined by occurrences of laboratory-acquired infections [5], [7]. VEEV has been weaponized in the past and is considered an important biodefense pathogen and select agent. Humans infected with VEEV manifest symptoms ranging from fever, headache, sore throat, malaise, myalgia, and vomiting to a severe neurological disease and coma [3], [5], [7]. Currently no therapeutics or vaccines have been FDA-approved for public use; however, the live attenuated strain, TC-83, is used as a vaccination for equines, military and at-risk personnel [1], [3], [5].

VEEV is an enveloped virus, ∼70 nm in diameter with a single-stranded positive sense RNA genome [3], [8], [9]. The viral genome is ∼11,400 nucleotides in length and encodes for 4 nonstructural proteins (nsP1–4) and 3 structural proteins (capsid, 6K, E1 and E2 envelope glycoproteins) [1], [8], [9]. The structural proteins initiate packaging and budding of virion particles from the surface of infected cells [8]. The membrane-associated nsP1 functions in RNA synthesis and capping [10], [11]. The viral protease, nsP2 cleaves the newly translated polyprotein into individual components and nsP4 functions as the RNA polymerase [10], [11]. Cytoplasmic replication for minus-strand synthesis early in infection serves as the template for plus-strand synthesis later in infection. This is facilitated by interaction of the nsPs with host factors [8]–[10]. Apart from its role in RNA synthesis, nsP3 has not yet been fully characterized [10], [11]. However, chimeric analysis and mutational studies implicated nsP3 as having a role in pathogenicity in mice [10]. nsP3 has 2 domains: a highly conserved N-terminal domain and a variable C-terminal domain as determined by amino acid sequence alignment of several alphaviruses [10]. Furthermore, it was found that nsP3 is phosphorylated on serine and threonine residues in the C-terminal end of the protein and that the hyperphosphorylated form may have a role in viral RNA synthesis [10].

The transcription factor, p65 regulates the expression of many target genes, which include genes that control cellular stress response, apoptosis, proliferation and cell adhesion as well as the innate and adaptive immune responses [12]–[16]. A variety of stimuli can activate the NF-κB response, such as cytokine stimuli (Tumor Necrosis Factor –α [TNF-α], Interleukin-1 [IL-1]), UV stress, DNA damage, lipopolysaccharide and virus infection, which result in p65 nuclear translocation and transcription regulation [12], [13], [15]–[21]. Upstream of the NF-κB cascade, adaptor proteins such as TNF-receptor-associated factors (TRAFs), MAP or ERK kinase kinase 3 (MEKK3) and TGF-β-activated kinase 1 (TAK1) are recruited to phosphorylate the IKK complex [14]–[20], [22]. The IKK complex is comprised of 3 subunits: IKKα, IKKβ and IKKγ [also referred to as NF-κB essential modulator (NEMO)] [12]–[14], [16]–[21]. This multi-protein complex is approximately 700–900 kDa and is deemed to be the “master coordinator of NF-κB activation” [13], [14], [17], [18], [20]. In the cytoplasm of an uninfected cell, NF-κB subunits are bound to inhibitory κB proteins (IκB) [12]–[20]. IκB proteins function by masking the nuclear localization sequence (NLS) found in a Rel-homology domain of p65, thus sequestering p65 in the cytoplasm [12]–[15], [19]. The activated IKKβ rapidly phosphorylates IκBα on S32 and S36, which is followed by IκBα ubiquitin-dependent proteasomal degradation and nuclear translocation of p65 [14]–[19].

Multiple viruses are known to influence the NF-κB cascade where critical steps in the cascade are hijacked to aid in viral replication. Several studies have implicated viruses and viral proteins as activators of the NF-κB cascade as well as serving as binding partners to proteins in the NF-κB pathway [12], [13], [17], [18], [20]. Viruses such as Human Immunodeficiency virus, Human T-Lymphotrophic virus, Ebstein Barr virus and Human herpesvirus 8 have been shown to not only activate the NF-κB signaling pathway, but also associate with the signaling components to enhance viral pathogenesis and viral life cycle [22]. Alternatively, viruses can indirectly interact with NF-κB by hijacking host proteins to stimulate activation and in so doing divert elements of the NF-κB pathway to amplify viral replication [22].

In the current manuscript, we determined that VEEV infection activated the NF-κB cascade as demonstrated by phosphorylation of p65 and IκBα as well as nuclear enrichment of p65. The upstream kinase that contributes to phosphorylation and nuclear translocation of p65 is the IKK complex. Macromolecular reorganization of IKKβ was observed in the event of VEEV infection, which was not observed in cells infected with a UV-inactivated virus. These data in conjunction with previous evidence of a similar reorganization of IKKβ in Rift valley fever virus (RVFV) infection [23] enabled us to hypothesize that IKKβ may play an important role in the replication of VEEV in host cells. Treatment of VEEV infection with documented inhibitors of IKKβ function such as BAY-11-7082, BAY-11-7085, and IKK2-IV revealed a decrease in virus replication. Animals infected with the TC-83 strain of VEEV, when treated with BAY-11-7082 demonstrated increased survival when compared to vehicle treated controls. To further reinforce the role of IKKβ in VEEV life cycle, we observed that VEEV replication was decreased in IKKβ^−/−^ cell lines; while when IKKβ was over-expressed, we observed an increase in viral output. Mass spectrometry analysis indicated that the VEEV protein nsP3 interacted with IKKβ which provides a possible mechanism for the increase in viral replication in our over-expression system. Therefore, our studies demonstrate a potential role for IKKβ in VEEV replication. This investigation may offer IKKβ as a prospective therapeutic target in down-regulating VEEV replication and provide insight into the host-viral interactions.

## Materials and Methods

### Ethics Statement

All animal experiments included in this manuscript were conducted in compliance with regulations by the Institutional Biosafety Committee and the Institutional Animal Care and Use Committee (approval #221) of George Mason University.

### Viruses and Cell Lines

The live-attenuated virus TC-83 used in this study was obtained from BEI resources. Eighty three passages of the virulent IAB Trinidad donkey (TrD) strain in guinea pig heart cells resulted in the TC-83 virus [24]. TrD and TC-83 genomes differ at 12 nucleotide positions [24]. TC-83 attenuation has been mapped to changes in the 5′-noncoding region and the E2 envelope glycoprotein [24]. The replication of TC-83 has been well studied both *in vitro* and *in vivo* and is a BSL-2 model for the fully virulent BSL-3 VEEV TrD. Experiments with TC-83 were performed under BSL2 settings and those with the wild type viruses were conducted under BSL3 requirements. Wild type Eastern Equine Encephalitis Virus (EEEV) GA97 was obtained from Dr. Jonathan Jacobs (MRIGlobal) and wild type Western Equine Encephalitis Virus (WEEV) (California 1930 strain) was obtained from ATCC. All select agents used in the manuscript are registered with the Centers for Disease Control and Prevention and conducted at George Mason University's Biomedical Research Laboratory, which is registered in accordance with Federal select agent regulations. As a control virus TC-83 strain was inactivated by exposure to ultraviolet radiation and termed UV-TC-83. UV inactivation of the virus was carried out using a Stratalinker UV crosslinker (model 1800). The inactivation was achieved by delivering an energy dose equivalent to 1200 µJoules X 100 per dose five times with a 2 minute interval between dosing.

Human astrocytoma cells (U87MG cells) and African Green Monkey kidney epithelial cells (Vero cells) were maintained in DMEM supplemented with 10% Fetal Bovine Serum (FBS), 1% Penicillin/Streptomycin and 1% L-Glutamine at 37°C, 5% CO_2_. Inhibitory κB kinase knockout (IKKβ^−/−^) and wild type mouse embryonic fibroblast (WT MEFs) cells were a kind gift from Dr. Cynthia Masison from NIH/NCI [25], [26]. IKKβ^−/−^ MEFs and WT MEFs were maintained in DMEM supplemented with 10% Fetal Bovine Serum (FBS), 1% Penicillin/Streptomycin and 1% L-Glutamine at 37°C, 5% CO_2_. Rat AP7 neuronal cells (a gift from Dr. Diann Griffin) were cycled at 33°C with 7% CO_2_ in DMEM supplemented with 10% FBS, 1% Penicillin/Streptomycin and 1% L-Glutamine. For differentiating the AP7 neuronal cells, the cycling media was modified with the addition of 1 µg/mL insulin, 20 µM dopamine and 100 µM ascorbic acid. The cells were then incubated at 39°C in 5% CO_2_ for 5 to 7 days for complete differentiation.

### Viral Infections

Cells were seeded in a 96-well plate such that confluency was attained the next day. The media was removed and saved and was referred to as conditioned media. The cells were infected for 1 hour to allow for viral adsorption at 37°C. The viral inoculum was removed and replaced with the conditioned media. The cells were incubated at 37°C, 5% CO_2_. The supernatant was collected 24 hours later and stored at −80°C until analyzed.

### Inhibitor Studies

Cells were seeded in a 96-well plate at a density of 10,000 cells per well. The next day the cells were pretreated with inhibitors, BAY-11-7082 (Sigma, Catalogue No. B5556), BAY-11-7085 (Sigma, Catalogue No. B5681), IKK2 compound IV (Santa Cruz Biotechnology, Catalogue No. sc-203083), 5,7-dihydroxy-4-methylcoumarin (DMC) (Santa Cruz Biotechnology, Catalogue No. sc-254863), *o*-phenanthroline (O-Phe) (Santa Cruz Biotechnology, Catalogue No. sc-202256) for 2 hours. The conditioned media (media containing inhibitor) was removed and viral infections proceeded at multiplicity of infection (MOI) of 0.1 for U87MGs and MOI: 1 for neuronal cells for 1 hour. The viral inoculum was removed and replaced with conditioned media. The cells were incubated for an additional 24 hours at 37°C, 5% CO_2_. The supernatants were collected and stored at −80°C until analyzed.

### Plaque Assay

Plaque assays were performed as described in earlier publications [23], [24]. Briefly, Vero cells were seeded at a density of 1×10^6^ cells per well in a 6-well plate. Viral supernatants, diluted in DMEM, were used to infect Vero cells in duplicate. The plates were incubated at 37°C, 5% CO_2_ for 1 hour with occasional rocking. A 3 ml overlay comprising 2× E-MEM and 0.6% agarose (1∶1) was added to each of the wells. Once solidified the plates were incubated for an additional 48 hours at 37°C, 5% CO_2_. The plaque assay was completed by the addition of a 10% Formaldehyde solution to the surface of the agarose plugs followed by 1 hour incubation at room temperature. The plates were then rinsed with diH_2_O and the agarose plugs removed. A 1% crystal violet solution containing 20% ethanol was added to each of the wells and incubated at room temperature with rocking for 30 minutes. The plates were rinsed with diH2O, visible plaques counted and the viral titers determined as PFU/mL.

### Cell Viability Assays

Cell viability was measured using a Cell-Titer-Glo Luminescent Cell Viability kit (Promega, Catalogue No. G7570) as per the manufacturer's instructions. Briefly, cells were seeded in 96-well white wall plates at 10,000 cells per well and incubated for 24 hours. Cells were treated with inhibitors and 24 hours post treatment cell viability was determined. Alternatively, cells were pre-treated with inhibitors for 2 hours and infected with TC-83 (MOI indicated in the associated figure legends) for 1 hour, after which the conditioned media was replaced and cells incubated for an additional 48 (for neurons) or 72 (for U87MGs) hours. To determine cell viability Cell-Titer-Glo reagent was added to the cells in a ratio of 1∶1. The plate was shaken for 2 minutes and incubated for 10 minutes at room temperature. Luminescence was detected using the DTX 880 multimode detector (Beckman Coulter).

### Plasmids

FLAG-IKKβ plasmid was a kind gift from Dr. Fengyi Wan (Johns Hopkins University). The WEEV_nsP3_V5 plasmid was a gift from Dr. David Miller (University of Michigan). VEEV pTC83 was a kind gift from Ilya Frolov of the University of Alabama at Birmingham. The pcDNA3.1_nsP3_HA was constructed using standard PCR recombination techniques. In brief, the nsP3 region of TC83 was subcloned out with primers containing a linker region (Ser-Ala-Ser-Ala) and HA-tag at the C-terminal end of nsP3. The last residue of nsP3 was mutated from an alanine to a valine to prevent nsP2 cleavage of the tag. The construct was confirmed with enzymatic digestion and sequencing. Plasmid and primer sequences are available upon request.

### Transfections and Protein Extracts

Transfections were performed using Attractene Transfection Reagent (Qiagen, Catalogue No. 301005) as per the manufacturer's instructions. As control plasmids, pUC19 or pcDNA3.1 was used. In preparation of whole cell lysates, the media was removed and the cells washed twice with PBS. Next the cells were lysed with lysis buffer that consisted of a 1∶1 mixture of T-PER reagent (Pierce, Catalogue No. 78510), 2× Tris-glycine SDS sample buffer (Invitrogen, Catalogue No. LC2676), 2.5% β-mercaptoethanol, and protease and phosphatase inhibitor cocktail (1× Halt mixture, Pierce). The collected cell lysates were boiled for 10 minutes and stored at −80°C until analyzed.

### Column Fractionation

The details of the column fractionation system have been extensively described previously [23], [24], [27]. Briefly, U87MG cells were seeded in T-150 flasks and incubated at 37°C, 5% CO_2_ until confluent. The cells were infected with TC-83 virus and UV-TC-83 at an MOI of 5. The cells were harvested 24 hours post-infection and pelleted by centrifugation (1,200 rpm for 10 minutes at 4°C). The cell pellets were lysed with lysis buffer comprising 50 mM Tris– HCl (pH 7.5), 120 mM NaCl, 5 mM ethylenediaminetetraacetic acid, 0.5% NP-40, 50 mM NaF, 0.2 mM Na3VO4, 1 mM DTT, and one complete protease cocktail tablet/50 mL. Two milligrams of total protein was subjected to chromatography using a Superose 6 HR 10/30 size-exclusion chromatography column and an AKTA purifier system (GE Healthcare, Piscataway, NJ, USA). For a better separation of small molecular weight complexes from fractions eluting off the far right side of the chromatogram, a quarter inch gap was introduced to the top of the Superose 6 column. A 1 ml loop was used to inject the sample into the system. Running buffer was set at a constant flow rate of 0.3 ml/minute and 0.5 ml fractions of the flow-through were collected at 4°C. A total of approximately 50 fractions were collected for each sample. The columns employed in this study were used for multiple runs prior to fractionation of the TC-83-infected or UV-inactivated control extracts, ensuring reproducible fractionation profiles. The 50 fractions collected range from ∼2.2 mDa to ∼100 kDa. These fractions were precipitated using 4 volumes of ice-cold 100% acetone. Lysates were centrifuged at 4°C for 10 minutes at 12,000 rpm, supernatants were removed, and the pellets were allowed to dry for a few minutes at room temperature. The pellets were resuspended in Laemmli buffer and analyzed by immunoblotting.

### Immunoprecipitation

Infection of U87MG cells with TC-83 virus and UV-TC-83 virus were conducted at an MOI of 5. Infected cells were maintained at 37°C, 5% CO_2_. Cells were pelleted and lysed in a buffer containing Tris-HCl (pH 218 7.5), NaCl (120 mM), EDTA (5 mM), NP-40 (0.5%), NaF (50 mM), Na3VO4 (0.2 mM), DTT (1 mM) and one tablet complete protease inhibitor cocktail per 50 ml. Cell lysis was performed on ice for 10 minutes, after which the lysates were centrifuged at 4°C for 10 minutes at 10,000 rpm. Supernatants were transferred to new tubes and protein quantitated by Bradford protein assay (BioRad, Hercules, CA, USA). Two milligrams of total protein was incubated overnight with rotation, at 4°C with normal rabbit IgG (Santa Cruz Biotechnology, Catalogue No, sc-2027) or α-IKKβ antibody (Santa Cruz Biotechnology, Catalogue No. sc-7329). A 30% slurry of Protein A+G beads (Calbiochem, Rockland, MA) was added to the IPs and incubated for 2 hours with rotation, at 4°C. The IPs were centrifuged briefly and beads were washed 1× with TNE150+0.1% NP-40, followed by a 2× wash with TNE50+0.1% NP-40.

### Western Blot Analysis

Whole cell or fractionated lysates were separated on a 4–20% Tris-Glycine Gel and transferred to a polyvinyl difluoride (PVDF) membrane using the iBlot gel transfer system (Invitrogen). The membranes were blocked in 1% dry milk in PBS-T at room temperature. Primary antibodies to VEEV Capsid (BEI Resources, NR 9403), VEEV Glycoprotein (BEI Resources, NR 9404), total p65 (Santa Cruz Biotechnology, Catalogue No. sc-7151), phospho-p65 (ser536) (Santa Cruz Biotechnology, Catalogue No. sc-33020), phospho-IκBα (Santa Cruz Biotechnology, Catalogue No. sc-21869), total IκBα (Santa Cruz Biotechnology, Catalogue No. sc-847), IKKα (Santa Cruz Biotechnology, Catalogue No. sc-7218), IKKβ (Santa Cruz Biotechnology, Catalogue No. sc-7329), IKKγ (Santa Cruz Biotechnology, Catalogue No. sc-8330), HA probe (F-7) (Santa Cruz Biotechnology, Catalogue No. sc-7392), V5 (AbD Serotec, Catalogue No. MCA 1360) and HRP conjugated actin (Abcam, Catalogue No. ab49900) were used according to manufacturer's instructions. The blots were incubated with respective primary antibody overnight at 4°C. Following 2 washes with PBS-T the blots were incubated with respective secondary HRP-coupled antibody. After 3 washes with PBS-T and 1 wash with PBS, the membranes were visualized by chemiluminescence using SuperSignal West Femto Maximum Sensitivity Substrate Kit (ThermoScientific) and a BIO-RAD Molecular Imager ChemiDoc XRS system (BIO-RAD).

### Immunofluorescence

U87MG cells were seeded at a density of 10,000 cells/well in an 8-well chambered slide. The cells were either mock treated, transfected or infected as described earlier. Cells were fixed with 3.7% paraformaldehyde for 20 minutes and permeabilized with 0.5% Triton X-100 in PBS for 15 minutes. Slides were washed with PBS and blocked at room temperature for 10 minutes with 3% BSA. The slides were incubated with primary antibody for 1 hour in the dark at 37°C. The samples were then washed three times with PBS and incubated with respective secondary antibody Alexa Fluor antibodies (Invitrogen) for 1 hour in the dark at 37°C. Slides were washed three times with PBS and incubated with DAPI for 10 minutes in the dark at room temperature. Following an additional PBS wash, the slides were mounted with Fluoromount G (SouthernBiotech, Catalogue No. 0100-01) and stored in the dark, at 4°C overnight. The cells were imaged using Nikon Eclipse TE2000-U. Images were taken at 60× magnification.

### Animal Studies

All experiments were carried out in biosafety level 2 (BSL-2) facilities and in accordance with the Guide for the Care and Use of Laboratory Animals (Committee on Care And Use of Laboratory Animals of The Institute of Laboratory Animal Resources, National Research Council, NIH Publication No. 86–23, revised 1996). C3H/HeN mice (n = 7 per group) were infected with TC-83 through the intranasal route at a concentration of 2×10^7^ PFU/mL. Animals were pretreated for one day and post treated once a day with BAY-11-7082 (10 mg/kg) through the subcutaneous route for 10 days. Survival was monitored up to a period of 2 weeks. Control mice were TC-83 infected and treated with DMSO (100% through subcutaneous injection). In each group, three animals were sacrificed at days 3, 7 and 10 post infection and virus titers were measured in both serum and brain by plaque assays. Mice were euthanized with CO_2_ and the serum was collected with 23 gauge needle right away, by cardiac puncture, 500–700 µl of blood was collected from each mouse, the blood was spun in the centrifuge for 5 minutes at 14000 rpm, the serum was collected, and viral plaque analysis was performed on serum. Mouse brains were collected from euthanized animals after the cardiac puncture. Collected brains were homogenized using IKA ULTRA-TURRAX Tube drive and DT-20 tube system, brains were homogenized in D-MEM supplemented media, and viral burden plaque analysis was performed.

### LC-MS/MS

LC-MS/MS analysis was carried out as previously described [23]. Briefly, samples were first lysed in 8M urea, after which, they were reduced using DTT and acetylated using iodoacetamide. The reduced and alkylated proteins were trypsin digested (Trypsin, Promega) for 4 hours at 37°C. The digested peptides were eluted using ZipTip purification (Millipore) and LC-MS/MS analysis of the peptides was performed by LTQ-tandem MS/MS equipped with a reverse-phase liquid chromatography nanospray (ThermoFisher). After sample injection, the column was washed for 5 minutes at 200 nl/min with 0.1% formic acid; peptides were eluted using a 50-minute linear gradient from 0 to 40% acetonitrile and an additional step of 80% acetonitrile (all in 0.1% formic acid) for 5 minutes. The LTQ- MS was operated in a data-dependent mode in which each full MS scan was followed by five MS-MS scans where the five most abundant molecular ions were dynamically selected and fragmented by collision-induced dissociation using normalized collision energy of 35%. Tandem mass spectra were matched against the National Center for Biotechnology Information mouse database by Sequest Bioworks software (ThermoFisher) using full tryptic cleavage constraints and static cysteine alkylation by iodoacetamide.

### Statistical Analysis

Individual experiments were performed in triplicate. Each experiment was performed as 3 independent experiments. Graphs are representative of the average of three independent experiments. Error bars (standard deviations) were calculated from the three independent experiments and are represented thusly. Unless otherwise stated, all statistical analyses was performed with the unpaired, two-tailed Student T-test using GraphPad's -QuickCalcs software, (GraphPad).

## Results

### Activation of the NF-κB Signaling Cascade in TC-83 Infected U87MGs

**Figure 1:**
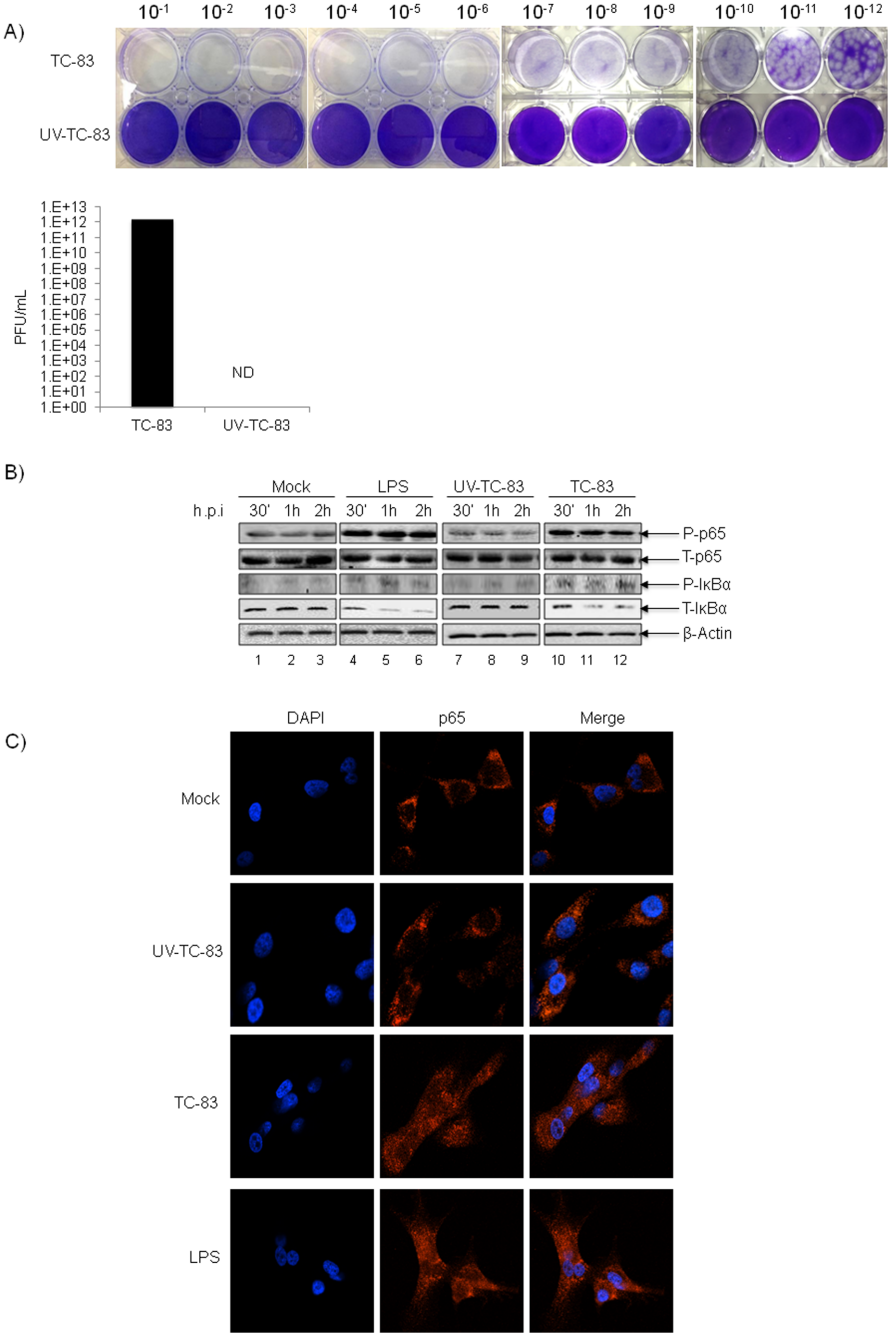
Activation of NF-κB complex in TC-83 infected cells. A) UV inactivation of the virus was carried out using a Stratalinker UV crosslinker (model 1800). The inactivation was achieved by delivering an energy dose equivalent to 1200 µJoules X 100 per dose five times with a 2 minute interval between dosing. UV-TC-83 and TC-83 were serially diluted and used to infect Vero cells. UV-TC-83 inactivation was confirmed by plaque assay. Plaques were photographed and counted 48 hours post-infection. Plaque counts are represented graphically. B) U87MG cells were either mock infected, treated with LPS (1 µg/mL) or infected with TC-83 or UV-TC-83 (MOI: 1). At 30 minutes, 1 and 2 hours post-infection cells were lysed and protein extracts were resolved by SDS-PAGE and subsequently immunoblotted with antibodies against phosphorylated p65 and phosphorylated IκBα. Total p65, total IκBα and β-actin served as controls. The western blot is representative of 2 independent experiments. C) U87MGs were either mock infected, treated with LPS (1 µg/mL) or UV-TC-83 or TC-83 infected (MOI: 3). One hour post-infection cells were fixed, probed with p65 antibody followed by incubation with Alexa-Fluor 568. The cells were stained with DAPI to observe the nuclei. Images were taken using Nikon Eclipse TE2000-U at 60× magnification and are representative of 2 independent experiments. ND = not detectable.

Recent microarray studies indicate an increase in inflammatory gene expression, an up-regulation of many Toll like Receptors (TLRs) along with their associated kinases, transcription factors and downstream target genes in VEEV infected mouse brain [28], [29]. These observations were strongly suggestive of a role for NF-κB in the *in vivo* pathology associated with VEEV infection. Therefore we investigated if infection with the live-attenuated strain of VEEV, TC-83 would result in activation of the NF-κB signaling cascade. Phosphorylation of IκBα on serine 32/36, p65 on serine 536 and p65 nuclear enrichment were used as markers of cascade activation. As a control, a UV-inactivated form of TC-83, termed UV-TC-83, was used. Inactivation of the UV-TC-83 virus was validated by plaque assays. As can be seen in Figure 1A, no plaques could be detected with the UV inactivated virus when compared to TC-83 at all dilutions examined. To determine if VEEV activates the NF-κB signaling cascade, U87MGs were either mock infected, treated with LPS (1 µg/mL) or infected with UV-TC-83 or TC-83 (MOI: 1) and at 30 minutes, 1 hour and 2 hours post-infection cells were lysed. Protein extracts were resolved by SDS-PAGE and immunoblotted with antibodies specific for phosphorylated p65 (Ser 536) and phosphorylated IκBα (Ser 32/36) (Figure 1B). As controls antibodies against total p65, total IκBα and β-Actin were used. Compared to UV-TC-83 infected cells, increased phosphorylation of p65 (Ser536) was observed in TC-83 infected cells as early as 30 minutes post-infection (Figure 1B, compare lanes 7 and 10). No apparent change was observed in the levels of total p65 in all the samples (Figure 1B). Increased degradation of IκBα was observed in TC-83 infected cells (which coincided with a modest increase in the phosphorylation of IκBα), which was not observed in UV-TC-83 infected cells (Figure 1B). This data indicated that TC-83 induced phosphorylation of both p65 at serine 536 and IκBα at serine 32/36 at early time points after infection. To further validate the activation status of p65, we performed confocal microscopy analysis to determine whether there was an increase in the nuclear pool of p65 in TC-83 infected cells. U87MGs were uninfected (mock), treated with LPS (1 µg/mL) or infected with UV-TC-83 or TC-83, fixed at 1 hour post-infection, incubated with p65 antibody and Alexa-Fluor 568 secondary antibody and subsequently stained with DAPI. As demonstrated by confocal microscopy analysis, in TC-83 infected cells we observed nuclear enrichment of p65 when compared to a more diffuse distribution of p65 observed in the control cells (Figure 1C). Nuclear enrichment of p65 therefore provided additional evidence of activation of the NF-κB cascade following infection by TC-83 strain of VEEV.

### Molecular Reorganization of the IKK Complex in TC-83 Infected Cells

**Figure 2:**
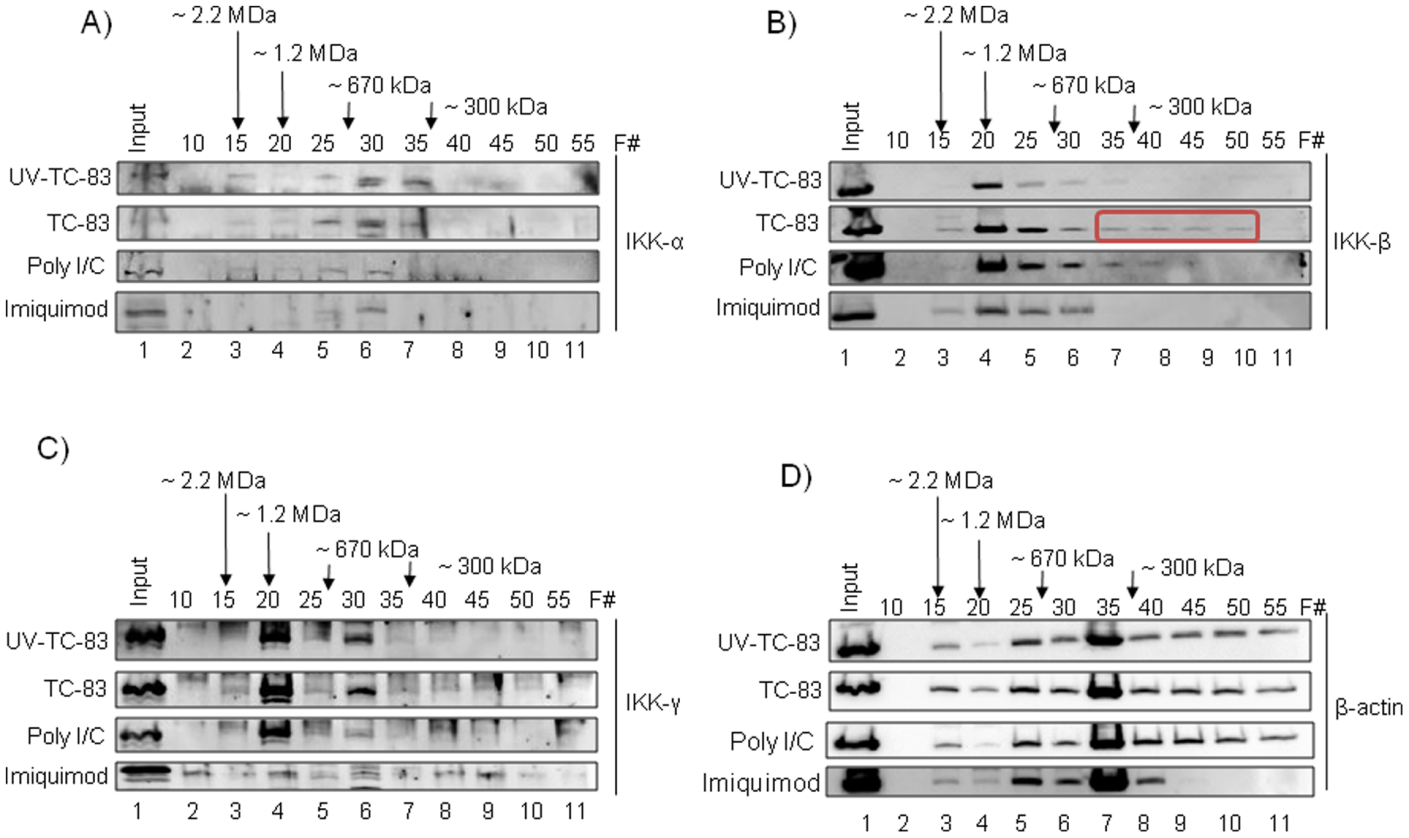
Macromolecular reorganization of the IKKβ complex in TC-83 infected cells. U87MG cells were infected with TC-83 or UV-TC-83 (MOI: 5) or the cells were treated with Poly I∶C (10 µg/mL) or Imiquimod (2 µg/mL). Cells were lysed 24 hours post-infection and post treatment and protein extracts were quantified. Two milligrams of total protein was subjected to chromatography using a Superose 6 HR 10/30 size-exclusion chromatography column and an AKTA purifier system. Every 5th fraction was acetone precipitated using 4 volumes of ice-cold 100% acetone and incubated for 15 minutes on ice. Lysates were centrifuged at 4°C for 10 minutes at 12,000 rpm, supernatants were removed, and the pellets were allowed to dry for a few minutes at room temperature. The pellets were resuspended in Laemmli buffer and analyzed by immunoblotting using IKKα (A), IKKβ (B), IKKγ (C) and β-actin (D) antibodies. Every fifth fraction ranging from 2.2 mDa to 100 kDa is represented on the western blot. Smaller IKKβ complexes (highlighted in the red box) in the higher fractions suggests a rearrangement of the IKKβ complex in TC-83 infected cells.

In the canonical NF-κB pathway, the kinase complex responsible for phosphorylation of IκBα and p65 (Serine 536) is the IKK complex [14]–[17], [20]. The IKK multi-protein complex (∼700–900 kDa) is comprised of 3 subunits: IKKα, IKKβ and IKKγ [12]–[14], [16]–[21]. We have previously demonstrated by size fractionation methods that in RVFV infected human cells, IKKβ underwent molecular reorganization leading to the formation of novel, low molecular weight forms of IKKβ which we had labeled IKKβ2 [23], [24], [27]. We hypothesize that this novel complex contains IKKβ and IKKγ. We had also published that IKKβ2 possessed kinase activity and could phosphorylate host targets including IκBα and viral proteins [23]. We asked whether IKKβ underwent a similar reorganization in the case of TC-83 infected cells. Here, U87MGs were infected with UV-TC-83 or TC-83 and total cell lysates were obtained 24 hours post-infection. Two milligrams of total protein was subjected to size-fractionation chromatography using a Superose 6 HR 10/30 size-exclusion chromatography column and an AKTA purifier system. The details of the procedure have been extensively described in previous publications [23], [24]. Every 5th fraction was resolved by SDS-PAGE followed by subsequent immunoblotting with antibodies for IKKα, IKKβ and IKKγ (Figure 2). In TC-83 infected cells, IKKβ was reorganized in comparison with the UV-TC-83 infected cells, with the appearance of low molecular weight complexes at approximately 200 kDa (Figure 2B red rectangle). These novel complexes are reminiscent of the IKKβ2 complex observed previously in the case of RVFV infected human cells [24]. IKKβ complex reorganization was not due to changes in total IKKβ as the input lanes in the TC-83 and UV-TC-83 lysates are comparable (Figure 2B, lane 1). The same extracts were immunoblotted with β-Actin, and as can be seen in Figure 2D there is an equivalent distribution of β-Actin in fractions 25–55 in all extracts (Figure 2D, lanes 5–11). No observable differences in the reorganization of the other IKK components – IKKα and IKKγ – between TC-83 and UV-TC-83 infected cells were detected (Figure 2A and 2C).

Sharma *et al.* observed a significant up-regulation of TLR3 and TLR7 gene expression levels as well as NF-κB dependent gene expression in VEEV-infected mice brains [28]. This prompted the question whether the IKKβ modulation was a TLR3 and/or TLR7 dependent mechanism. U87MGs were treated with the TLR3 agonist, Poly I∶C and the TLR7 agonist, Imiquimod at concentrations of 10 µg/mL and 2 µg/mL respectively. Poly I∶C and Imiquimod recognize dsRNA and ssRNA respectively. Total cell lysates were obtained at 24 hours post treatment. Two milligrams of total protein was subjected to chromatography as described above. Every 5th fraction was resolved by SDS-PAGE followed by subsequent immunoblotting with antibodies for IKKα, IKKβ and IKKγ (Figure 2). Interestingly, Poly I∶C treated U87MGs showed an IKKβ distribution profile similar to that seen in TC-83 infected cells (Figure 2B, lanes 4–9) although the lower molecular weight complexes of IKKβ were more prominent in the TC-83 infected cells. The IKKα and IKKγ components did not undergo any noteworthy reorganization in the event of Poly I∶C treatment between the TC-83 infected and UV-TC-83 infected cells (Figure 2A and 2C). Imiquimod treatment did not display significant changes in any of the IKK components. In fact, while Imiquimod treatment did not alter total protein distribution (data not shown), it resulted in reorganization of β-actin (Figure 2D). These results suggested that the reorganization and activation of IKKβ in VEEV infected cells was possibly at least in part a TLR3-based mechanism. Taken together, these data suggested that TC-83 infection of U87MGs induced a TLR3 dependent macromolecular reorganization of IKKβ.

### Inhibition of IKKβ Decreases VEEV Replication

**Figure 3:**
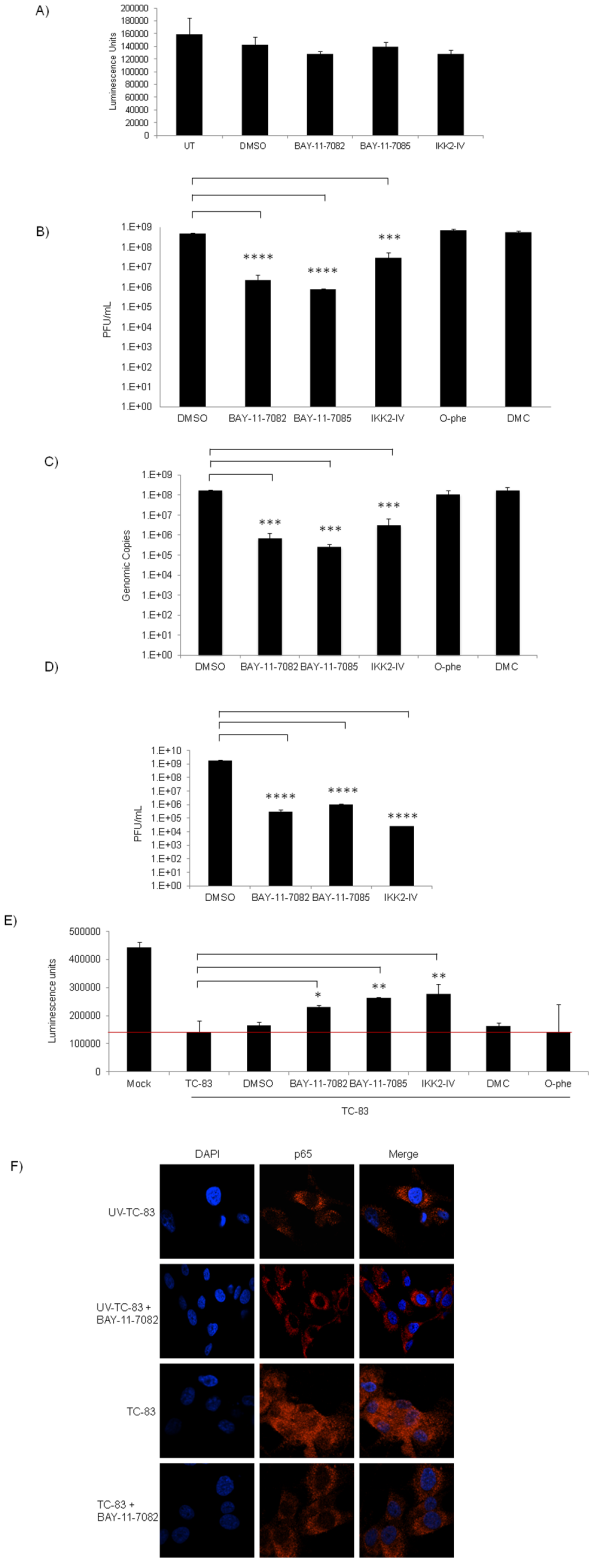
IKKβ inhibitors decrease viral load in TC-83 infected cells pre and post exposure. A) U87MGs were untreated, DMSO treated or pretreated with IKK inhibitors (1 µM) for 2 hours and 24 hours later cell viability was measured using the Cell-Titer-Glo Luminescent Cell Viability Assay. B) U87MG cells were pretreated with IKK inhibitors (1 µM), BAY-11-7082, BAY-11-7085 and IKK2-IV and non-IKK specific inhibitors (1 µM), O-Phe and DMC for 2 hours. The conditioned media (media containing inhibitor) was removed and viral infections proceeded (MOI: 0.1) for 1 hour. The viral inoculum was removed and replaced with the conditioned media. The cells were incubated for an additional 24 hours. The supernatants were collected from infected and inhibitor treated cells. Infectious viral titers were determined by plaque assay. O-phe and DMC served as positive control inhibitors. C) Supernatants from B) were subjected to q-RT-PCR analysis to determine viral RNA copies using VEEV specific primers. D) U87MGs were infected at MOI: 0.1 for 1 hour and then treated with BAY-11-7082 (1 µM), BAY-11-7085 (1 µM) and IKK2-IV (1 µM) 4 hours post-infection. Supernatants were collected 24 hours post-infection and viral titers were determined by plaque assay. E) U87MGs were pretreated with IKK inhibitors (1 µM) and non-IKK inhibitors (1 µM) for 2 hours and followed by a 1 hour infection. Conditioned media was replaced and cell viability assay was performed 72 hours later using the Cell-Titer-Glo Luminescent Cell Viability Assay. The red line is representative of the base line for luminescence units, where luminescence units above this value are indicative of increased cell viability. F) U87MGs seeded in an 8-well chambered glass slide were either pre-treated with DMSO or BAY-11-7082 (1 µM) for 2 hours and then infected with UV-TC-83 or TC-83 (MOI: 0.1) for 1 hour. The conditioned media was replaced after the infection. One hour post-infection the cells were fixed and probed for p65 with subsequent incubation with Alexa Fluor 568. The cells were stained with DAPI to observe the nuclei. Images were taken using Nikon Eclipse TE2000-U at 60× magnification and are representative of 2 replicate samples within the same experiment. The graphs represent an average of 3 independent experiments. Error bars for the 3 independent experiments were calculated and are represented thusly. **** p≤0.0001, *** p≤0.005, ** p≤0.01 and * p≤0.05.

To characterize the role of IKKβ in TC-83 infection, well documented inhibitors of IKKβ function namely, BAY-11-7082, BAY-11-7085 and IKK2-compound IV (IKK2-IV) were employed in the subsequent experiments [30]–[32]. A study conducted by Keller *et al.*, showed that in Karposi sarcoma-associated herpesvirus infected primary effusion lymphoma cells, low doses of BAY-11-7082 effectively inhibited phosphorylation of IκBα [33]. IKK2-IV inhibits IKKβ and thus interferes with NF-κB activation. We wanted to investigate whether IKKβ function was required for VEEV replication in infected cells. As determined by Cell-Titer-Glo assay, the inhibitors were found not to be cytotoxic to U87MGs when compared to the DMSO treated cells and untreated cells (UT) (Figure 3A). Along with the IKK inhibitors, 2 additional inhibitors were employed, *o*-phenanthroline (O-Phe) and 5,7-dihydroxy-4-methylcoumarin (DMC). These inhibitors (O-Phe and DMC) interfere with DNA binding and transcriptional activation by p65 [24]. U87MGs were pre-treated with the panel of inhibitors for 2 hours. The conditioned media was removed and the cells were infected with TC-83. One hour following infection, the conditioned media was added back onto the cells and 24 hours post-infection the supernatants were collected. Controls included infected cells treated with DMSO alone. Plaque assays were conducted on the collected supernatants to analyze for infectious virus. Treatment with BAY-11-7082 and IKK2-compound IV reduced viral titers by approximately 2 logs when compared to the DMSO control (Figure 3B). Treatment with BAY-11-7085 resulted in a decrease in infectious viral particles by approximately 3 logs when compared to the DMSO control (Figure 3B). In contrast, treatment with O-Phe or DMC did not affect viral titers. We next determined if the observed inhibitory effect on TC-83 observed in Figure 3B was a consequence of infection-defective virus or low levels of released virus. Using the same supernatant as that in Figure 3B, quantitative-RT-PCR (q-RT-PCR) studies were conducted with VEEV-specific primers. As shown in Figure 3C, BAY-11-7082, BAY-11-7085 and IKK2-IV treatment significantly inhibited TC-83 genomic copies when compared to the mock infected and DMSO infected controls. This result mirrored that observed in Figure 3B. We then determined whether these inhibitors could effectively inhibit viral replication when introduced following virus exposure. Four hours after infection with TC-83, the cells were treated with BAY-11-7082, BAY-11-7085 and IKK2-compound IV. Supernatants were collected 24 hours post-infection and analyzed by plaque assay. As shown in Figure 3D, post exposure treatment of infected cells with IKKβ inhibitors, BAY-11-7082, BAY-11-7085 and IKK2-compound IV, demonstrated a down-regulation of extracellular virus by 4, 3 and 5 logs respectively when compared to the DMSO control. This data indicated that treatment with IKK inhibitors resulted in a decrease in TC-83 genomic copies and infectious viral particles, suggestive of a requirement for an activated IKK complex to enhance TC-83 replication (Figure 3B, C and D).

We hypothesized that inhibition of viral replication may also result in an increase in survival of the host cell. Therefore, we determined whether treatment of TC-83-infected cells with the IKKβ inhibitors would increase survival of infected cells. To this end, U87MGs were either untreated uninfected (Mock) or cells were infected and treated with the inhibitors or the DMSO control. The cells were incubated for 72 hours after which viability was measured using the Cell-Titer-Glo Luminescent Cell Viability Assay. The red line is representative of a base line for luminescence units, values above which are indicative of increased cell viability. BAY-11-7082, BAY-11-7085 and IKK2-compound IV statistically increased cell viability when compared to the TC-83 infected cells (Figure 3E). O-Phe and DMC did not display any protective effects on cell viability when compared to the TC-83 infected and DMSO controls (Figure 3E). To further demonstrate the efficacy of the IKK inhibitor, BAY-11-7082, we chose to investigate localization of p65 in the infected and treated cells. In Figure 3F, confocal microscopy illustrates that p65 does not enrich in the nuclei of infected cells upon treatment with BAY-11-7082. Cumulatively, these data suggested that the IKK inhibitors were efficacious in inhibiting TC-83 replication in U87MGs.

### Inhibition of IKKβ in Neuronal Cells Decreases VEEV Replication

**Figure 4:**
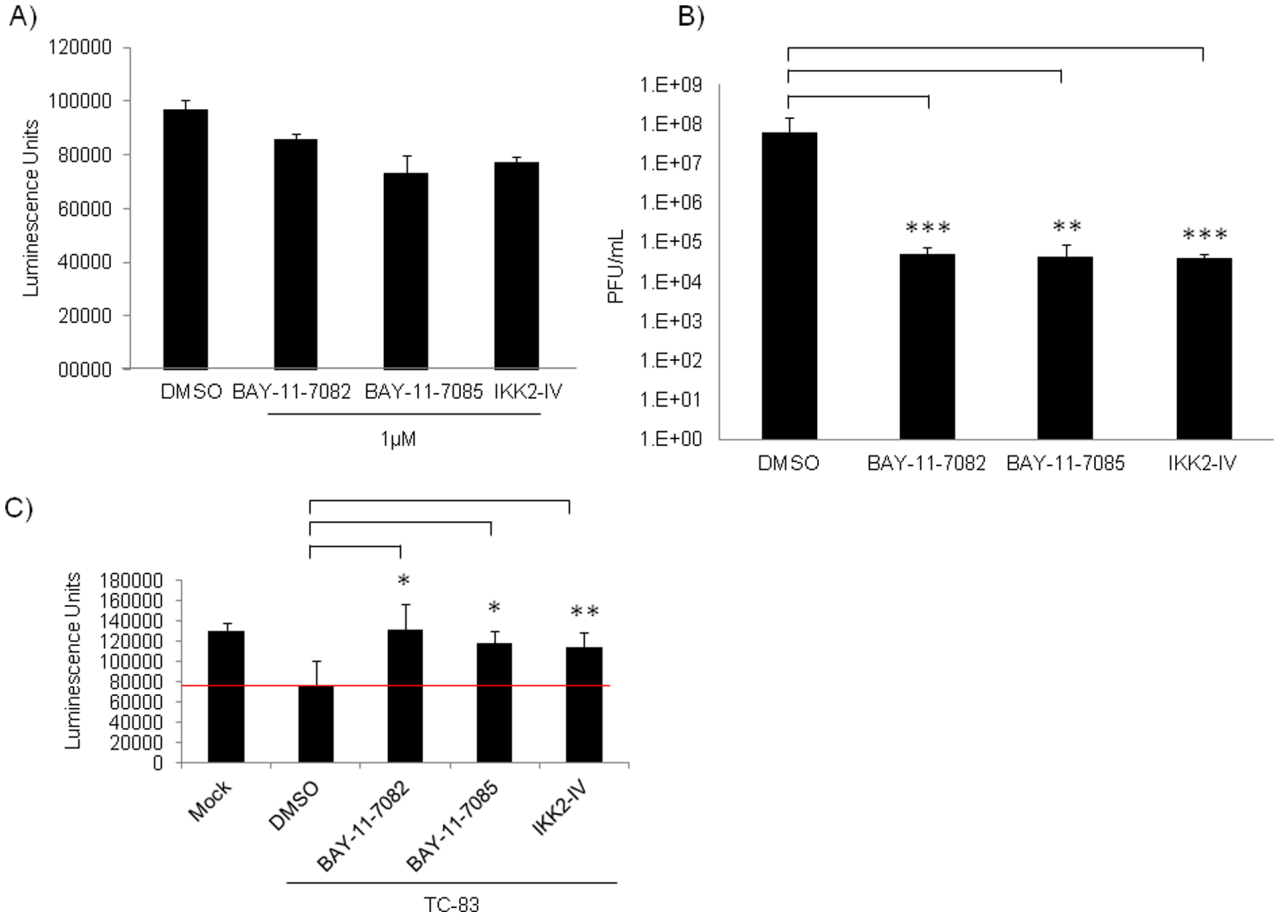
IKKβ inhibitors decrease TC-83 replication in rat AP7 neuronal cells. A) Neurons were pre-treated with DMSO or with IKK inhibitors (1 µM) for 2 hours and 24 hours later cell viability was measured using the Cell-Titer-Glo Luminescent Cell Viability Assay. B) Neurons were pretreated with IKK inhibitors (1 µM), BAY-11-7082, BAY-11-7085 and IKK2-IV for 2 hours. Following the pretreatment, the conditioned media (media containing inhibitor) was removed and the cells infected at MOI: 1 for 1 hour. The viral inoculum was removed and the conditioned media replaced. Supernatants were collected 24 hours post-infection, and infectious viral titers were determined by plaque assay. C) Neurons were pretreated with IKK inhibitors (1 µM) for 2 hours and infected with TC-83 for 1 hour. Conditioned media was replaced after removal of the viral inoculum. Cell viability assay was performed 48 hours later using the Cell-Titer-Glo Luminescent Cell Viability Assay. The red line is representative of the base line for luminescence units, such that luminescence units above this are indicative of increased cell viability. The graphs are representative of 2 independent experiments. Error bars for the independent experiments were calculated and are represented thusly. *** p≤0.005, ** p≤0.01 and * p≤0.05.

We next wanted to investigate if the IKK complex in neuronal cells is required for TC-83 replication. First, IKK inhibitors namely, BAY-11-7082, BAY-11-7085 and IKK2-compound IV were tested for toxicity in rat AP7 neuronal cells. As determined by Cell-Titer-Glo assay, the inhibitors were found not to be cytotoxic to neuronal cells when compared to the DMSO treated cells (Figure 4A). To determine if the IKK complex is required for TC-83 replication in neurons, the cells were pre-treated with the IKK inhibitors for 2 hours. The conditioned media was removed and the cells were infected with TC-83. One hour following infection, the conditioned media was added back onto the cells and 24 hours post-infection the supernatants were collected. Controls included infected cells treated with DMSO alone. Plaque assays were conducted on the collected supernatants to analyze for infectious virus. Treatment with IKK inhibitors reduced viral titers by approximately 3 logs when compared to the DMSO control (Figure 4B). We next determined whether TC-83-infected neurons treated with the IKKβ inhibitors would increase survival of the infected cells. Neurons were either uninfected and untreated (Mock) or infected and treated with the inhibitors (1 µM) or the DMSO control. The cells were maintained for up to 48 hours after which viability was measured using the Cell-Titer-Glo Luminescent Cell Viability Assay. The red line is representative of a base line for luminescence units, and values above this base line are indicative of increased cell viability. BAY-11-7082, BAY-11-7085 and IKK2-compound IV statistically increased cell viability of infected cells when compared to the DMSO infected controls (Figure 4C). Taken together, these data suggest that in neuronal cells IKKβ has a role in TC-83 replication.

### IKKβ Inhibitor Treatment Down-Regulates Wild Type VEEV In Vitro

**Figure 5:**
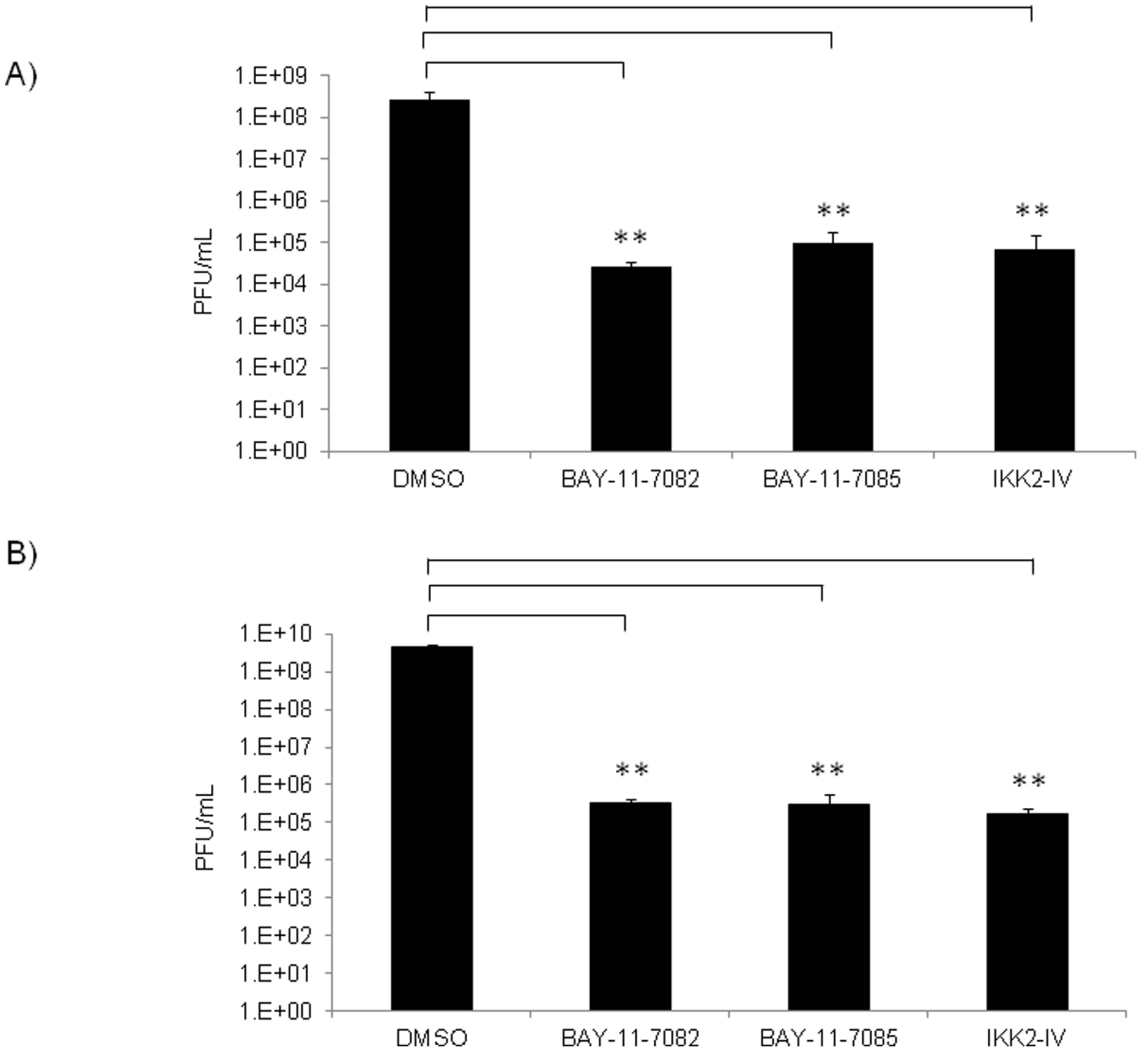
IKKβ inhibitors are effective in decreasing viral load of wild type VEEV. A) U87MG cells (A) or neuronal rat AP7 cells (B) were pretreated with 1 µM IKK inhibitors, BAY-11-7082, BAY-11-7085 and IKK2-IV for 2 hours. The cells were infected with the wild type strain of VEEV (TrD) at a MOI: 0.1 (A) or MOI: 1 (B) for 1 hour. The conditioned media (media containing inhibitor) was removed prior to the viral infections and replaced after the viral inoculum was removed. The cells were incubated for an additional 24 hours. The supernatants were collected from all samples and viral titers were determined by plaque assay. The graphs are representative of 2 independent experiments. Error bars (Standard deviations) for 3 replicates within the 2 independent experiments were calculated and are represented thusly. ** p≤0.01.

We wanted to determine whether inhibition of IKKβ had any consequence on replication of the wild type Trinidad Donkey (TrD) strain of VEEV. U87MGs were pre-treated with BAY-11-7082, BAY-11-7085 and IKK2-IV for 2 hours. The cells were infected with TrD for 1 hour. The infected cells were incubated for a further 24 hours before the supernatants were collected and analyzed for infectious viral particles by plaque assay. Figure 5A represents the viral titers for TrD after BAY-11-7082, BAY-11-7085 and IKK2-compound IV treatments in U87MGs. All three inhibitors reduced viral replication when compared to the DMSO control. We next wanted to determine if the inhibitors would exert a similar effect in neurons. To this end, rat AP7 neurons were pretreated with 1 µM BAY-11-7082, BAY-11-7085 and IKK2-compound IV and then infected with TrD for 1 hour. Neurons are not readily infected with TC-83 and hence a higher MOI was used for the infection (personal communication, Dr. Kehn-Hall). Plaque assays indicated that all of the inhibitors demonstrated a decrease in viral replication compared to the DMSO control (Figure 5B). In summary, the results demonstrate that inhibition of IKKβ function resulted in inhibition of TrD (virulent) strain of VEEV.

### BAY-11-7082 Decreases Wild Type Alphavirus Replication

**Figure 6:**
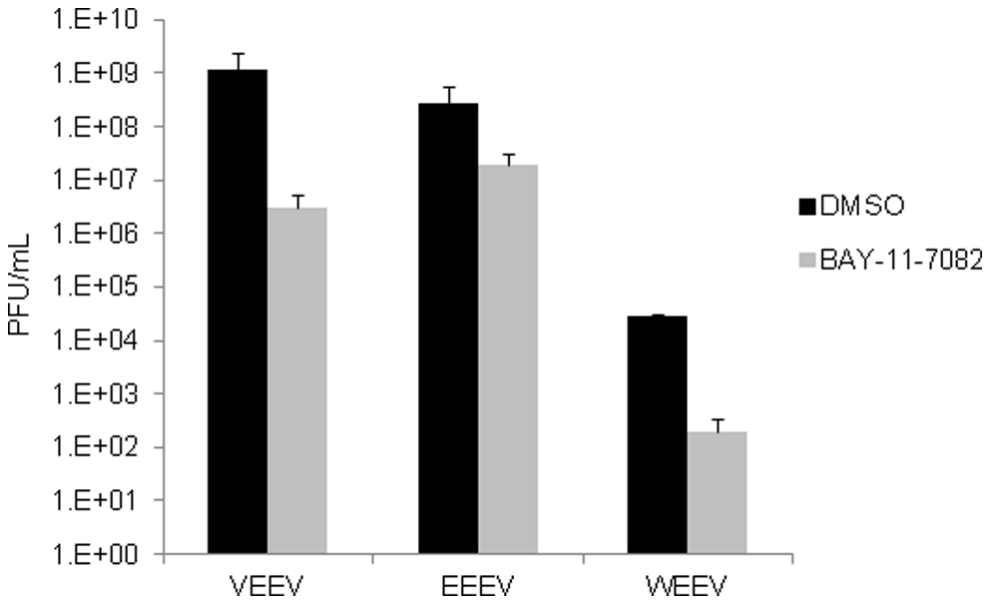
BAY-11-7082 decreases replication of wild type alphaviruses. U87MGs were pre-treated with DMSO or BAY-11-7082 (1 µM) for 2 hours. The conditioned media (media containing inhibitors) was removed and the cells infected with VEEV TrD, EEEV GA97 and WEEV (California 1930 strain) for 1 hour at an MOI of 0.1. The conditioned media was replaced and the supernatants collected 24 hours later. Plaque assays were performed to determine viral titers. The graph is the average of n = 3 from a single experiment. Error bars for 3 replicates were calculated and are represented thusly.

The IKK inhibitor, BAY-11-7082 has demonstrated to be effective at decreasing VEEV replication. This prompted the next question: does BAY-11-7082 produce a similar decrease in replication of other New World alphaviruses? To this end, cells were pretreated with DMSO or BAY-11-7082 and then infected with VEEV TrD, EEEV strain GA97 and WEEV (California 1930 strain). All infections were performed for 1 hour. Conditioned media was replaced and the supernatants were collected 24 hours later. Plaque assays were performed to determine infectious viral particles. Figure 6 demonstrates that BAY-11-7082 decreased viral replication of TrD and WEEV (California 1930 strain) by 3 logs compared to the DMSO control. Interestingly, BAY-11-7082 decreased replication of EEEV GA97 by 1 log when compared to the DMSO control. This result suggested that the IKK inhibitor, BAY-11-7082 decreased replication of all 3 alphaviruses tested.

### Inhibitor Treatment Decreases TC-83 Viral Load *In Vivo*

**Figure 7:**
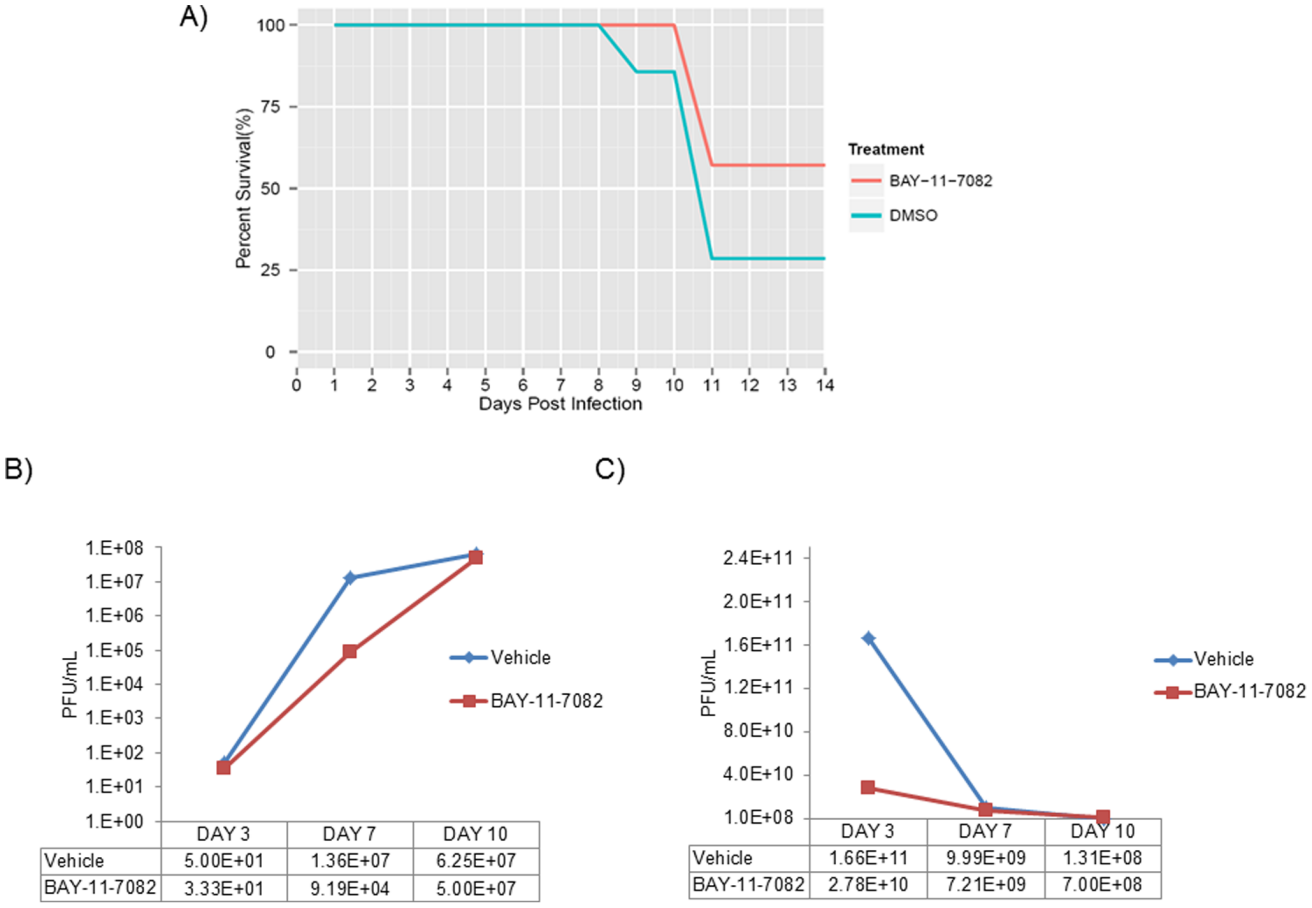
Increased survival in TC-83 infected mice when treated with BAY-11-7082. C3H/HeN mice (n = 7 per group) were infected with TC-83 by the intranasal route (2×10^7^ PFU/mL). Animals were pretreated for one day and post treated once a day with BAY-11-7082 (10 mg/kg) subcutaneously for 10 days. Control mice were TC-83 infected and treated with DMSO. A) Survival was monitored up to a period of 2 weeks. Results from one experiment are represented by a line graph for comparison of survival of the two groups over days of observation. B) Serum samples were collected from 3 animals per group on days 3, 7, and 10. Viral titers were determined by plaque assay. Results are shown in a line graph to indicate differences in viral titers over time. C) Homogenized brains were collected in parallel with serum samples. Plaque assay results of those samples are indicated by a line graph to indicate differences in viral titers over time. Values were obtained by averaging numbers from three brain and serum samples at each time point.

The above *in vitro* studies demonstrated BAY-11-7082 as a strong inhibitor of both the live-attenuated strain, TC-83 and the wild type strain TrD. We next wanted to determine if BAY-11-7082 could have a protective effect *in vivo*. C3H/HeN mice (n = 7 per group) were infected with TC-83 through the intranasal route at a concentration of 2×10^7^ PFU/mL. Animals were pretreated for one day and post treated once a day with BAY-11-7082 (10 mg/kg) administered subcutaneously. Survival was monitored up to a period of 2 weeks. Control mice were TC-83 infected and treated with DMSO control. Figure 7A shows the data plotted as percentage survival over the 14 day period. The trends show a divergence between the inhibitor treatment group and the control mice. At days 3, 7, and 10 samples from 3 animals per group were taken. Plaque assays were performed to determine infectious viral titers in the serum samples, as shown in Figure 7B. On day 3, the average of treated serum samples were 66.7% that of the control mice serum samples. On day 7, a 3 log reduction in infectious viral titers was observed in the treated serum samples than in the control serum samples. Treated serum samples had an average of 80% that of the control samples at day 10. Homogenized brains were collected in parallel with serum samples. Infectious viral titers in the brain homogenates of treated and untreated mice were determined by plaque assay. From Figure 7C, BAY-11-7082 treated mice had a 1 log reduction in the infectious viral titers when compared to the control mice (compare 2.78×10^10^ PFU/mL with 1.66×10^11^ PFU/mL respectively). Therefore, the inhibitor maintained viral loads in the brain to only 16% that of the control mice until day 3. The differences between infectious viral titers of treated and untreated brain homogenates became relatively less pronounced as the study progressed. This could be due to difficulty of the BAY-11-7082 compound to traverse the blood brain barrier *in vivo*. While this experiment provides supportive *in vivo* evidence of the role of IKKβ in virus replication, it also provides opportunities to consider development of second-generation derivatives that may be more proficient in passing through the blood brain barrier.

### A Requirement for IKKβ in TC-83 Replication in U87MGs

**Figure 8:**
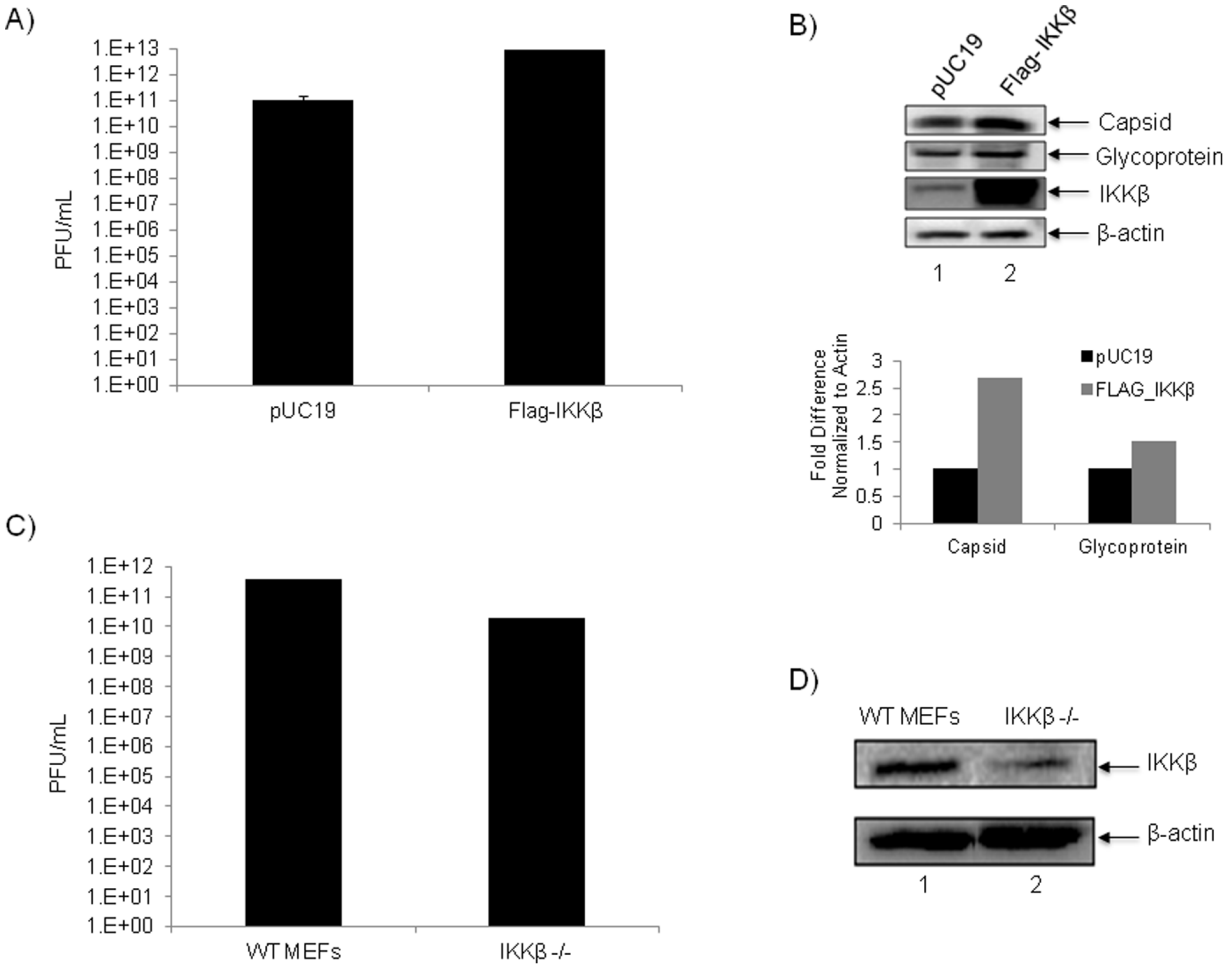
A requirement for IKKβ in TC-83 replication *in vitro*. A) U87MG cells were transfected in triplicate with 1 µg of FLAG-IKKβ and 1 µg of pUC19 as a control plasmid for a total of 48 hours. Transfected cells were then infected with TC-83 (MOI: 0.1). Supernatants were collected 24 hours post-infection and plaque assays were performed to determine viral titers. B) Total protein from the cell lysates in A) were subjected to western blot and probed for VEEV capsid, VEEV glycoprotein, IKKβ and β-Actin. FLAG_IKKβ and pUC19 protein bands were quantified using Image J software and normalized to β-Actin. Average fold differences of 2 independent experiments are depicted graphically. C) Mouse embryonic fibroblasts (MEFs) lacking IKKβ (IKKβ^−/−^) and wild type (WT) MEFs were infected with TC-83 (MOI: 0.1) in triplicate. Supernatants were collected 24 hours post-infection and plaque assays were performed to determine viral titers. D) Western blot analysis of WT MEFs and IKKβ^−/−^ confirmed depletion of IKKβ^−/−^ in the knockout cell line when probed for IKKβ. β-actin served as a loading control. The graphs represent an average of 3 independent experiments. Error bars for the 3 independent experiments were calculated and are represented thusly.

Our data thus far is indicative of an important role for IKKβ in the VEEV life cycle leading us to hypothesize that over expression of IKKβ in infected cells may contribute to better viral replication while a depletion of IKKβ may result in an inverse phenotype. Therefore, to further validate the relevance of IKKβ to TC-83 replication, a FLAG-tagged version of IKKβ (FLAG-IKKβ) was over-expressed in U87MGs, the cells were infected with TC-83 and the number of infectious viral particles was determined by plaque assay. U87MGs were transfected in triplicate with 1 µg of either FLAG-IKKβ or pUC19 (which served as our control vector) for a total of 48 hours. The transfected cells were infected with TC-83 and 24 hours post-infection supernatants were collected and the infectious viral titers were determined by plaque assay (Figure 8A). Over expression of IKKβ resulted in an approximate 2 log increase in TC-83 replication when compared to the control cells (Figure 8A). Cell extracts from the same samples above were subjected to western blot analysis to investigate if the increase in viral titers correlated with an increase in VEEV capsid and glycoprotein expression. The same blot was reprobed to validate over expression of IKKβ when compared with pUC19-transfected cells. β-Actin served as a loading control (Figure 8B). The band intensities of 2 independent experiments were quantified using Image J and the fold differences normalized to β-Actin and are represented graphically for pUC19 and FLAG_IKKβ infected cells. This result suggested that IKKβ over expression led to an increase in the number of infectious viral particles. The opposite experiment was performed to validate the requisite for IKKβ in TC-83 replication. We infected wild type mouse embryonic fibroblasts (WT MEFs) and IKKβ^−/−^ mouse embryonic fibroblasts (IKKβ^−/−^ MEFs) with TC-83. Supernatants were collected 24 hours post-infection and plaque assays were performed. We observed that in the absence of IKKβ there is a reduction of ∼1.5 logs in the number of infectious viral particles in the TC-83 infected cells when compared to the infected WT MEFs (Figure 8C). Whole cell extracts of IKKβ^−/−^ MEFs and WT MEFs were resolved by SDS-PAGE and immunoblotted for IKKβ to verify the cell line was deficient in IKKβ protein production (Figure 8D). Collectively, our data supported our hypothesis that IKKβ played an important role in VEEV replication.

### The Interaction of IKKβ with VEEV nsP3

**Figure 9:**
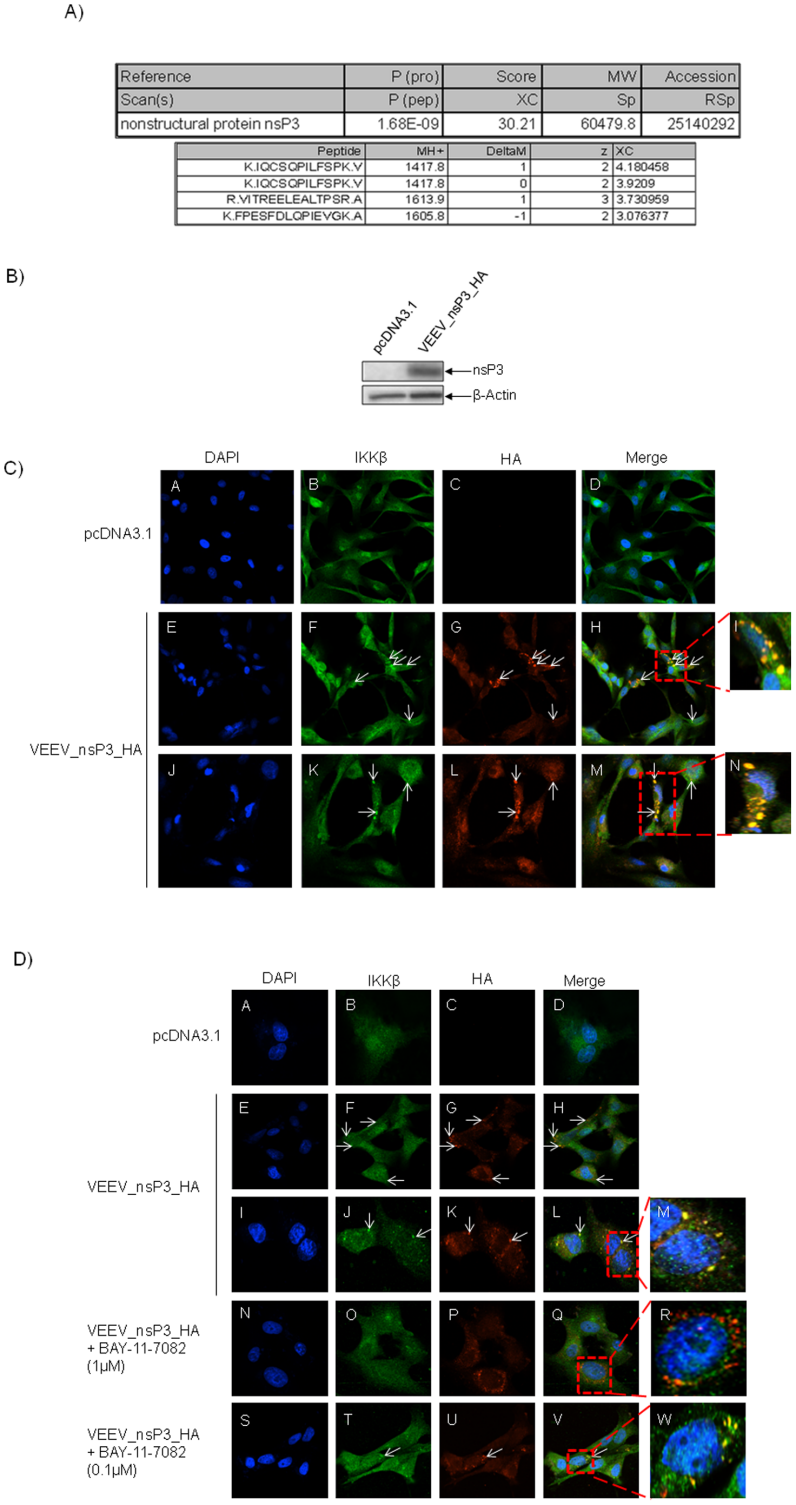
VEEV nsP3 interaction with host IKKβ. A) IKKβ was immunoprecipitated from VEEV infected U87MGs and Liquid Chromatography Tandem Mass Spectrometry (LC-MS/MS) was performed. Control immunoprecipitations were performed with an IgG antibody. Mass spectrometry analysis revealed that the viral nonstructural protein nsP3 interacted with IKKβ. B) U87MGs were transfected in a 6-well plate with 5 µg of pcDNA3.1 and VEEV_nsP3_HA for 24 and 48 hours. Cell lysates were resolved using SDS-PAGE and subsequently immunoblotted with HA and β-actin served as a loading control. C) U87MGs were transfected in duplicate with (0.2 µg) VEEV_nsP3_HA and pcDNA3.1 (control), cells were fixed after 24 hours and probed with antibodies against the endogenous IKKβ and the HA tag. Cells were subsequently incubated with appropriate secondary Alexa Fluor antibodies and the nuclei stained with DAPI. Co-localization of IKKβ with nsP3 (yellow) was observed as shown by the arrows. The co-localization was confirmed by Z-stack analysis. Panels E–H and J–M serve as examples of transfected cells in a given field of view that show co-localization of IKKβ and VEEV_nsP3_HA at 24 hours post transfection. Panels I and N are magnified images of the outlined cells in red boxes in panels H and M respectively. Co-localization was found to be approximately in 71% of cells (72 cells were counted of which 55% demonstrated expression of nsP3. Of those cells that expressed nsP3, 71% showed co-localization of both IKKβ and VEEV_nsP3_HA proteins). D) U87MGs were transfected in duplicate with (0.2 µg) VEEV_nsP3_HA and pcDNA3.1 (control); cells were treated with BAY-11-7082 (1 µM and 0.1 µM). The cells were fixed 24 hours post transfection and probed with antibodies against the endogenous IKKβ and the HA tag. Cells were subsequently incubated with appropriate secondary Alexa Fluor antibodies and the nuclei stained with DAPI. Co-localization of IKKβ with nsP3 (yellow) was observed as shown by the arrows. The co-localization was confirmed by Z-stack analysis. Panels E–H and I–L serve as examples of transfected cells in a given field of view that show co-localization of IKKβ and VEEV_nsP3_HA at 24 hours post transfection. Panel M is a magnified image of the outlined cells in red boxes in panel L. Panels N–Q and S–V are examples of transfected and treated cells in a given field of view. Panels R and W are magnified images of the outlined cells in red boxes in panels Q and V respectively. Images were taken using Nikon Eclipse TE2000-U at 60× magnification and are representative of 3 independent experiments.

To determine a possible mechanism for the interaction between IKKβ and VEEV, we investigated if any of the viral proteins interacted with IKKβ. To that end, we immunoprecipitated IKKβ from VEEV infected U87MGs and performed Liquid Chromatography Tandem Mass Spectrometry (LC-MS/MS). Control immunoprecipitations were performed with an IgG antibody. Mass spectrometry analysis revealed that the viral nonstructural protein nsP3 interacted with IKKβ (Figure 9A). To validate this finding, an expression clone that contained an HA tag at the C-terminal end of TC-83_nsP3 was constructed: VEEV_nsP3_HA. An HA tag was utilized because the tag would allow for detection of nsP3 by western blotting and confocal microscopy. U87MGs were transfected with pcDNA3.1 (control plasmid) or VEEV_nsP3_HA in a 6-well plate for 24 hours. Cell extracts were prepared and the protein resolved by SDS-PAGE. The subsequent immunoblot indicated that VEEV_nsP3_HA expressed efficiently at 24 hours (Figure 9B). In an 8-well chambered slide, U87MGs were transfected with VEEV_nsP3_HA or with pcDNA3.1 (control). Twenty-four hours after transfection, cells were fixed and probed with IKKβ and HA. Co-localization of IKKβ with nsP3 was observed at 24 hours post transfection as shown by the arrows in Figures 9C. The co-localization was confirmed by Z-stack analysis and was quantified to be approximately in 71% of VEEV_nsP3_HA expressing cells (72 cells were counted of which 55% demonstrated expression of nsP3. Of those cells that expressed nsP3, 71% showed co-localization of IKKβ and VEEV_nsP3_HA). Figure 9C, panels E–H and J–M serve as examples of transfected cells in a given field of view that show co-localization of IKKβ and VEEV_nsP3_HA at 24 hours post transfection. Figure 9C, panels I and N are magnified images of the outlined cells in red boxes in panels H and M respectively. Foy *et al.* had recently shown that the nsP3 proteins of both Old World and New World alphaviruses form distinct complexes *in vitro* [2]. In that study the authors' analysis of the proteomics of the complex revealed proteins that have a prior history of association with IKKβ (For example: Hsp70 is known to associate with the IKK complex and potentially influence NEMO dependent complex oligomerization particularly under conditions of cellular stress; 14-3-3 proteins are known to associate with the IKK complex and influence ASK1 function during stress signaling) suggesting that these structures that form and grow in the presence of nsp3 may include large IKKβ containing complexes that are unique to stressed cells. [2]. To validate the co-localization and specificity of nsP3_HA and IKKβ, confocal microscopy was performed, whereby, U87MGs were transfected with nsP3_HA and pcDNA3.1 (control) for 24 hours and treated with BAY-11-7082 at 6 hours post transfection. The cells were fixed 24 hours post transfection and processed as described above. Figure 9D demonstrates that co-localization between nsP3_HA and IKKβ can be interrupted with BAY-11-7082 in a dose dependent manner. In Figure 9D, panels E–H and I–L serve as examples of transfected cells in a given field of view that show co-localization of IKKβ and VEEV_nsP3_HA at 24 hours post transfection. Panel M is an enlarged image of L showing the co-localization of VEEV_nsP3_HA and IKKβ. Panels N–Q and S–V are images of cells treated with BAY-11-7082 at 1 µM and 0.1 µM concentrations respectively. Panels R and W represent enlarged images of the cells in red boxes in panels Q and V respectively. Interestingly, the nsP3_HA complexes still form; however co-localization with IKKβ is interrupted with the higher treatment of BAY-11-7082. At the lower dose of BAY-11-7082, we observed co-localization of nsP3_HA with IKKβ. This result indicated that phosphorylation of IKKβ is required for the nsP3_HA- IKKβ interaction. Taken together, confocal microscopy has identified a potential interaction between the VEEV viral protein nsP3 and the host IKKβ protein at 24 hours post transfection, which is interrupted with BAY-11-7082 treatment.

### IKKβ Interacts with WEEV nsP3

**Figure 10:**
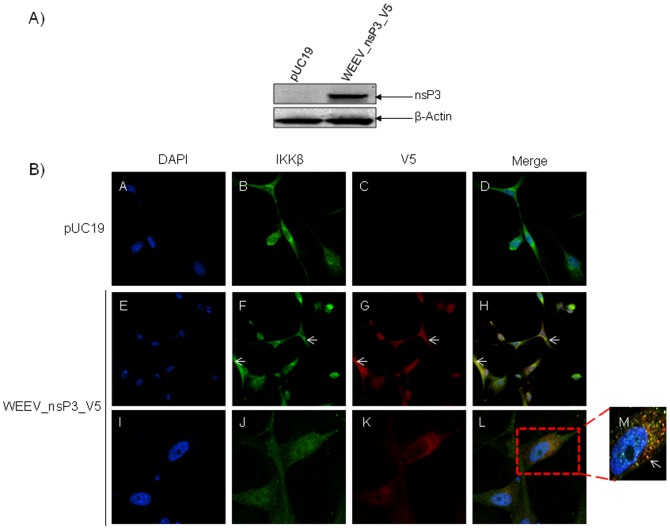
WEEV nsP3 interaction with host IKKβ. A) U87MGs were transfected in a 6-well plate with 5 µg of pUC19 and WEEV_nsP3_HA for 24 hours. Cell lysates were resolved using SDS-PAGE and subsequently immunoblotted with V5 antibody and β-actin served as a loading control. B) U87MGs were transfected with WEEV_nsP3_V5; cells were fixed after 24 hours and stained with antibodies against the endogenous IKKβ and the V5 tag. Cells were incubated with appropriate secondary Alexa Fluor antibodies and the nuclei stained with DAPI. Co-localization of IKKβ with WEEV_nsP3_V5 (yellow) was observed as shown by the arrows. B) Panels E–H serve as an example of transfected cells in a given field of view that show co-localization of IKKβ and WEEV_nsP3_V5 24 hours post transfection. Panels I-L represent magnified images of other cells showing co-localization of IKKβ and WEEV_nsP3_V5. Panel M is a magnified image of panel L. The co-localization was confirmed by Z-stack analysis. Co-localization was calculated to be approximately in 61% of cells (163 cells were counted of which 44% demonstrated expression of nsP3. Of those cells that expressed nsP3, 61% showed co-localization of both proteins). Images were taken using Nikon Eclipse TE2000-U at 60× magnification and are representative of 2 independent experiments.

The next question asked was: does this interaction occur with nsP3 of other alphaviruses? Here, U87MGs were transfected with a V5-tagged version of WEEV nsP3 (WEEV_nsP3_V5) for 48 hours. Sequence analysis of WEEV nsP3 displayed approximately 81% sequence similarity between the nsP3 proteins of VEEV and WEEV (data not shown). Cell lysates were collected and the protein extracts resolved by SDS-PAGE. The resultant western blot probed with V5 demonstrated expression of WEEV_nsP3_V5 at 24 hours post transfection (Figure 10A). We performed confocal analysis to determine whether IKKβ co-localized with nsP3 (Figure 10B). U87MGs were transfected with the WEEV_nsP3_V5 plasmid; cells were fixed after 24 hours and stained with antibodies against the endogenous IKKβ and the V5 tag. We observed co-localization of IKKβ with nsP3 as shown by the arrows in Figure 10B. The co-localization was confirmed by Z-stack analysis and was quantified to be approximately in 61% of cells (163 cells were counted of which 44% demonstrated expression of nsP3. Of those cells that expressed nsP3, 61% showed co-localization of both proteins). Taken together this data suggested that there is a potential interaction between nsP3 of VEEV and WEEV with the host IKKβ protein.

## Discussion

Encephalitic Old World alphaviruses such as Chikungunya virus and New World alphaviruses including VEEV cause diseases in many parts of the world and is primarily characterized by a rapid and explosive host inflammatory response. While the underlying mechanisms of inflammation are important for the host to mount an immune response to the virus, the inflammation is also often associated with debilitating outcomes such as neurodegeneration as has been demonstrated in the case of VEEV [34]. Alphaviral infections including VEEV do not currently have approved therapeutic options and although not fatal in humans, leads to debilitating disease. Our approach has been to identify changes in host responses to VEEV exposure and determine if those alterations are protective host responses or changes orchestrated by the virus to enable its own replication. Defining host changes that are critical to viral replication will be helpful in the development of host-based therapeutics to treat alphavirus infections.

Inflammatory responses are intimately linked to activation of the NF-κB signaling cascade. The NF-κB signaling cascade is responsive to signals from TLRs many of which have been shown as being activated in the case of VEEV infection of mouse models [28]. Our preliminary experiments had indicated that the NF-κB cascade was activated at very early times after infection by the canonical pathway. Previous studies with bunyavirus infections demonstrated that the activation of the canonical pathway that is IKKβ-dependent was linked to a macromolecular reorganization of the IKKβ protein complex [23], [24], [27]. This reorganization manifested as novel low molecular weight complexes of IKKβ that were unique to infected cells. Additional studies had demonstrated that the reorganization process was not a generic consequence of a viral infection as infection by a DNA virus did not elicit a similar response under comparable conditions. Generic activation of the NF-κB response by inflammatory mediators such as TNF-α also did not elicit the reorganization of IKKβ further underscoring the idea that this was a specific virus infection dependent event. Previous data suggested that the newly formed complexes were kinase active and phosphorylated viral targets [24]. In this manuscript, we report that IKKβ reorganized to form lower molecular weight versions similar to our observation with bunyaviruses (Figure 2). Collectively, our data based on IKKβ profiles under diverse conditions have led us to believe that this mechanism may be a process relevant to RNA virus infections. Our observation that treatment of cells with a TLR3 agonist (Poly I∶C) induces a comparable change in IKKβ fractionation profile while treatment with a TLR7 agonist (Imiquimod) did not indicated to us that the mechanism was dependent on the presence of cytoplasmic double stranded RNA molecules. With regard to VEEV infections, we infer that the IKKβ reorganization was a consequence of active viral replication, which triggers TLR3 activation. This inference is further supported by the fact that infection by a UV-inactivated virus does not trigger the IKKβ phenotype.

It is well known that viral proteins utilize host proteins to enhance their replication and suppress the innate immune response to evade recognition. We speculate that the alterations in kinase complexes such as IKKβ may alter their substrate specificities in a manner that may encourage phosphorylation of viral targets or decrease their ability to differentiate between a host and a viral target protein. Nevertheless, inhibition of such kinases that undergo reorganization in a manner that is unique to infected cells identifies novel candidates for host-based therapeutics. Inhibition of IKKβ function by well-documented inhibitors such as BAY-11-7082, BAY-11-7085 and IKK2-IV induced robust inhibition of both TC-83 and TrD strains in U87MGs (astroglioma cells) and in neurons (Figures 3, 4 and 5). BAY-11-7082 displayed inhibition in TrD, EEEV (GA79) and WEEV (California 1930 strain) replication (Figure 6). C3H/HeN mice treated with BAY-11-7082 demonstrated a protective phenotype when the animals were infected with the TC-83 strain of VEEV under conditions where 75% of infected animals die within 2 weeks (Figure 7). The fact that inhibition of the functionality of IKKβ inhibited viral replication while inhibition of the downstream NF-κB-dependent activation of transcription (DNA binding) did not affect the virus strongly suggested that the mechanism of inhibition was not a generic defect in transcription. This further emphasized the possibility that one or more viral proteins might be phosphorylated by IKKβ and this phosphorylation may be critical to enhance viral replication.

Our proteomic studies have identified the VEEV nsP3 protein as a potential interacting component with IKKβ in infected cells (Figure 9). Among the alphavirus nonstructural proteins, relatively little is known about nsP3 function. nsP3 is a phosphoprotein with no inherent enzymatic activity. A wide range of activities are associated with nsP3 that relate to enhanced viral replication in infected cells including negative strand RNA synthesis, viral packaging, initiation of translation, and alteration of the intracellular milieu of infected cells to down regulate the innate immune response while the exact mechanism behind these functions remains unknown. nsP3 from multiple alphaviruses have an extent of similarity in amino acid sequence while generic functionality of domains of the protein appears similar. More specifically, our BLAST analysis of nsP3 sequences from VEEV and from all alphaviruses indicated that there is about 60% similarity among all alphaviruses and about 81% similarity between VEEV strains. To arrive at this estimate, we performed our BLAST analysis with VEEV nsP3 (NP_740698.1) using the blastp algorithm (NCBI) against the NR database at NCBI, returning the top 5000 hits, but otherwise using default BLAST parameters (Expect threshold = 10, word size 3, BLOSUM 62 similarity matrix, a gap penalty of 11 to open and 1 to extend). There were 148 hits to VEEV nsP3 proteins, 833 hits to alphavirus nsP3 proteins. While the N-terminus of the protein is fairly conserved, the C-terminus houses a hypervariable domain (HVD), which appears to have accumulated changes during the course of evolution. However, many of these changes do not appear to affect function of the protein with regard to viral replication. The switching of HVDs between different alphaviruses exhibited subtle differences in the localization of the chimeric proteins in comparison with the respective parent proteins [2]. In the case of Sindbis virus, specific mutations in the nsP3 protein have been shown to decrease neurovirulence in mice drawing relationships between nsP3 and VEEV-induced neuronal phenotypes. The HVD of nsP3 is thought to be the mode by which nsP3 interacts with cellular proteins which will have distinct consequences. Sequence analysis of VEEV nsP3 revealed a close match to the consensus IKK phosphorylation sequence (DSΨXXS) between amino acids 380 and 386. The VEEV sequence was almost identical to the IKKβ phosphorylation site on the canonical host IκB substrates with the addition of one amino acid in the VEEV sequence. While we did not observe this consensus sequence in the EEEV nsP3 protein (which is missing the C-terminal portion of the nsP3 sequence altogether), we noticed a similar consensus in the Sindbis virus nsP3 protein between amino acids 370 and 375. In our studies, a WEEV nsP3 protein also demonstrated strong association with IKKβ by confocal microscopy studies. Therefore, it is possible that some of the alphavirus nsP3 proteins may be phosphorylated by the IKKβ enzyme. It remains to be determined whether this phosphorylation event is functionally comparable between the different alphaviruses in influencing viral replication.

In conclusion, our data have demonstrated that VEEV infection results in activation of the NF-κB cascade and reorganization of the host IKKβ enzymatic complex. Inhibition of IKKβ results in a robust inhibition of viral replication *in vitro* and *in vivo*. Proteomic analysis have identified that the viral protein nsP3 may interact with IKKβ. Future studies are aimed at dissecting the interaction between VEEV-nsP3 and IKKβ to determine effects of phosphorylation on viral replication.
